# NIR-Triggered and ROS-Boosted Nanoplatform for Enhanced Chemo/PDT/PTT Synergistic Therapy of Sorafenib in Hepatocellular Carcinoma

**DOI:** 10.1186/s11671-022-03729-w

**Published:** 2022-09-20

**Authors:** Chonggao Wang, Xiaolan Cheng, Hao Peng, Yewei Zhang

**Affiliations:** 1 Medical School, Southeast University, Nanjing, 210009 China; 2 Nanjing Hospital of Chinese Medicine, Nanjing, 210000 China; 3 School of Chinese Medicine & School of Integrated Chinese and Western Medicine, Nanjing University of Chinese Medicine, Nanjing, 210023 China; 4 Hepatopancreatobiliary Center, The Second Affiliated Hospital of Nanjing Medical University, Nanjing, 210009 China

**Keywords:** Sorafenib, Chemo/photothermal (PTT)/photodynamic (PDT) therapy, Hepatocellular carcinoma, Ferroptosis, P-glycoprotein (P-gp)

## Abstract

Although being the first-line treatment of advanced hepatocellular carcinoma (HCC), sorafenib (SOR) outcome is limited due to drug resistance and low tumor accumulation. Herein, with MnO_2_ as photothermal agent and chlorine6 (Ce6) as photosensitizer, a tumor-targeting and NIR-triggered multifunctional nanoplatform loading sorafenib (MnO_2_-SOR-Ce6@PDA-PEG-FA, MSCPF) was constructed. Owing to oxygen generator MnO_2_, MSCPF could generate excessive ROS, thus can alleviate tumor hypoxia and improve sorafenib accumulation in cancer cells. Besides, ROS production further strengthens Ce6-mediated PDT and PDA-mediated PTT. By exploiting these features, MSCPF exhibited excellent antitumor effects on HCC in the in vitro and in vivo studies, compared to solo sorafenib or PDT/PTT treatment. Further mechanism experiments suggested that MSCPF could inhibit P-gp expression and induce ferroptosis via deactivation of GPX4 and SLC7A11, which ultimately enhanced the antitumor efficacy of SOR. In summary, our work highlights a promising NIR-triggered and ROS-boosted nanoplatform for enhanced chemo/PDT/PTT synergistic therapy of SOR in HCC treatment.

## Introduction

Hepatocellular carcinoma (HCC) is the third leading cause of cancer-related death worldwide, with an overall 5-year survival rate less than 10% [1]. Despite promising and considerable advances in HCC diagnosis, over 50% of HCC patients are still at an advanced stage once diagnosed. The current therapy options for HCC include surgical resection, trans-arterial embolization, radiotherapy, and chemotherapy. However, for patients with advanced HCC, radical surgery is not amenable, and the only feasible curative strategy is chemotherapy [2]. Sorafenib (SOR), acting as the first FDA-approved multiple-target tyrosine kinase inhibitor (TKI), is currently an effective first-line systematic treatment agent for advanced HCC patients [3]. By targeting Raf-1, B-Raf, and tyrosine kinase activity such as PDGF, FLT-3, and VEGF-2, sorafenib not only can inhibit tumor growth but also exerts an antiangiogenic effect [4]. However, only one-third of advanced HCC patients benefit from sorafenib, and extension in life span after sorafenib treatment is very limited, with the median OS from 7.9 to 10.7 months [5]. This unsatisfactory partial response of sorafenib is associated with its notable drug resistance, low tumor accumulation, and limited tumor penetration [4, 6]. Herein, it is an urgent need to explore the in-depth mechanism of drug resistance, and design novel and effective therapeutic strategy to improve sorafenib efficacy in HCC.

Multidrug resistance (MDR) mostly associates with enhanced efflux of cytotoxic chemotherapeutic agents, which diffuse through the plasma membrane into cancer cells. Drug extrusion from cells is mediated by the ATP-binding cassette (ABC) multidrug efflux transporters such as P-glycoprotein (P-gp; ABCB1; MDR1) [7, 8]. Growing evidence showed that MDR efflux transporters were over-expressed in various drug-resistant cancer cell lines and MDR tumor specimens [9, 10]. These proteins could reduce the effective intracellular accumulation of chemotherapeutics in an ATP-dependent manner and thus remarkably compromised the antitumor efficacy. Beside MDR efflux transporters, hypoxia-driven pH decrease in the tumor environment also can lead to the protonation of weak-base drugs, thereby inhibiting their diffusion into cancer cells [11]. Ferroptosis is regarded as a novel from iron-dependent cell death driven by lipid peroxidation [12]. Recently, ferroptosis has been demonstrated to contribute to sorafenib resistance in human HCC cells [13]. YAP/TAZ and ATF4 drive sorafenib resistance by inhibiting ferroptosis, shedding new light on overcoming HCC therapy resistance [14]. At present, many studies have focused on the development of chemosensitizers or ABC transporters inhibitors to reverse MDR or improve the sensitivity of MDR cancer cells to chemotherapy [15]. However, the chemosensitizers and ABC transport inhibitors exhibited only modestly positive or even negative effectiveness in overcoming MDR, due to their nonspecificity, high concentrations to inhibit activity, undesirable pharmacokinetic interactions with chemotherapeutics, or formulation problems such as low solubility and permeability [16]. Novel strategies and platforms to enhance chemotherapy efficacy are highly desirable.

Recently, combination therapy that integrate chemotherapy with other therapeutic modalities have exhibited competitive advantages in overcoming chemoresistance [17–19]. Photodynamic therapy (PDT), a noninvasive treatment modality that uses photosensitizers and light to generate cytotoxic molecular species such as ROS to induce cell death, has received widespread attention in drug-resistant cancer treatment. By increasing intracellular ROS levels, PDT can directly photodegrade ABC transporter proteins (e.g., P-gp) and then promotes drug delivery into cancer cells [20]. Additionally, the intelligent nanodelivery system can control the release of drugs and then improve the distribution of chemodrugs in tumor tissues. For photothermal therapy (PTT), hyperthermia during treatment not only promotes the delivery of anticancer drug to deep tumor tissues by enhancing the permeability of tumor vascular, but also increases the sensitivity of drug-resistant cancer cells to chemotherapeutic drugs via repressing the expression of MDR-related efflux transporters [21, 22].

Herein, a multifunctional nanoplatform was assembled by utilizing manganese dioxide (MnO_2_) nanoparticles as photothermal agent and nanocarrier to deliver sorafenib and photosensitizer chlorine6 (Ce6). After subsequently coating with poly(ethyleneglycol)-folate (PEG-FA)-modified polydopamine (PDA), a new type of nanoscale chemo/PDT/PTT trimode platform (MnO_2_-SOR-Ce6@PDA-PEG-FA, denoted as MSCPF) was formed. The photothermal agent PDA not only prevents premature release of the loaded molecules, but also facilitates the post-modification of PEG and FA. With large mesoporous structures, MnO_2_ nanospikes show ultrahigh loading efficienciesis and widespread application in biomedicine [23]. In acidic tumor microenvironment, MnO_2_ collapses and then triggers Ce6 and sorafenib release. Furthermore, MnO_2_ could convert the endogenous hydrogen peroxide (H_2_O_2_) into oxygen (O_2_). The generated O_2_ alleviates oxygen-depleted environment in the tumor, which enhances ROS production to strengthen PDT. By exploiting these features, MSCPF nanoplatforms were expected to achieve synergized chemo/PDT/PTT therapy and excellent antitumor effect in cancer treatment.

## Materials and Methods

### Materials

KMnO_4_ (≥ 99%) (223,468, sigma), dopamine hydrochloride (DA·HCl, 98%) (1,225,204, sigma), hydrogen peroxide (H_2_O_2_, 30%) (349,887, sigma), polyvinyl pyrrolidone (PVP, 98%, Mw = 8,000 Da) (P110608, Aladdin), formamide (F7503, sigma) (99%), and 9,10-anthracenediyl-bis(methylene)dimalonic acid (ABDA) (75,068, sigma) were purchased from Sigma-Aldrich. Chlorin e6 (Ce6, 98%) (R-GMJCe6, Ruixi) was supplied by Xi’an Ruixi Biological Technology. Ethanol (≥ 99.7%) (10,009,218, Sinopharm) and dimethyl sulfoxide (DMSO, > 99.8%) (30,072,418, Sinopharm) were purchased from Sinopharm Chemical Reagent Company. Sorafenib (SOR, 99.5%) (S125098, Aladdin), FA-PEG-SH (*M*_w_ = 3,400 Da, 95%) (F163751, Aladdin) and HO-PEG-SH (*M*_w_ = 3,400 Da, 95%) (T164376, Aladdin) were supplied by Shanghai Aladdin Chemistry Company. Aqueous dilutions were prepared with deionized H_2_O. All other chemicals and reagents were commercially available and were used as received.

### Materials Characterization

Transmission electron microscopy (TEM; HT7800, Hitachi, acceleration voltage = 120 kV) was applied to characterize the structure of different nanoparticles. UV–vis spectra were measured with a PerkinElmer Lambda 750. The sizes and zeta potentials of variety nanoparticles were determined with a Malvern Zetasizer (Zetasizer Nano ZS, Malvern, UK). Dissolved O_2_ was measured with an oxygen probe (JPBJ-608, Shanghai REX Instrument Factory). Thermal images were captured with an IR camera (FOTRIC 225 s), and confocal images were recorded by a confocal laser scanning microscopy (Olympus IX 70 inverted microscope). The flow cytometry assay was carried out using a flow cytometer (Guava easyCyteTM).

### Synthesis of MnO_2_ Nanoparticles (MnO_2_ NPs)

MnO_2_ NPs were synthesized according to the reported method with some modifications [24]. Specifically, 0.5 g KMnO_4_ and 2 mL formamide were sequentially added to 200 mL PVP solution (5 mM in H_2_O), and the solution were stirred for 12 h. Then, the as-produced MnO_2_ NPs were separated by centrifugation at 13,000 rpm for 10 min and washed with H_2_O for three times.

### Synthesis of MnO_2_-SOR NPs

Sorafenib (SOR) loading into MnO_2_ was achieved by adding 20 mL SOR (0.5 mg/mL in DMSO) into 40 mL MnO_2_ NPs under ultrasonic condition. After 30 min, the solution is stirred for 48 h. Then, MnO_2_-SOR NPs (MS NPs) were separated by centrifugation at 12,000 rpm for 10 min and washed with H_2_O for three times.

### Synthesis of MnO_2_-SOR-Ce6@PDA

Ce6 loading was executed by adding 20 mL Ce6 (0.5 mg/mL in Tris–HCl, pH = 8.5) into 40 mL MnO_2_-SOR NPs under vortexing, and the solution was allowed to stirring for 48 h. Then, 10 mg of dopamine hydrochloride was added, followed by stirring for 4 h at room temperature. The final product, denoted MnO_2_-SOR-Ce6@PDA (MSCP), was centrifuged at 10,000 rpm for 10 min and washed three times with H_2_O to remove self-nucleated PDA nanoparticles.

### Synthesis of MnO_2_-SOR-Ce6@PDA NPs

The MSCP NPs and HS-PEG-FA were well dispersed in Tris–HCl buffer (pH = 8.5) and stirred for 12 h in the dark. Then, the mixture was centrifuged and washed several times with H_2_O to remove the remaining HS-PEG-FA. The final product, MnO_2_-SOR-Ce6@PDA-PEG-FA (MSCPF), was dispersed in H_2_O.

### Photothermal Effect Test

Different samples (H_2_O, MnO_2_, and MnO_2_@PDA) were irradiated with an 808 nm laser for 10 min at a power density of 1.5 W cm^−2^ to investigate the impacts of MnO_2_ and PDA on the photothermal performance. To examine the concentration dependency of photothermal effect, MnO_2_@PDA with different concentrations of 50, 100, and 200 μg mL^−1^ was irradiated for 10 min. Then, 0.5 W cm^−2^, 1.0 W cm^−2^, 1.5 W cm^−2^, and 2.0 W cm^−2^ were employed to investigate the influence of power density on photothermal performance. MnO_2_@PDA solution (200 μg mL^−1^) was irradiated with five on–off cycles with an 808 nm laser at a power density of 1.5 W cm^−2^ to study of the photothermal stability.

### Calculation of Photothermal Conversion Efficiency

The heating–cooling curve of MSCPF (200 μg mL^−1^) was recorded with an 808 nm laser at power density of 1.5 W cm^−2^ for 6 min. The photothermal conversion efficiency (PCE) was calculated according to the published method [25].

### Release of Sorafenib

The release of sorafenib (SOR) from the MSCPF NPs was monitored by UV–vis spectroscopy. To investigate the influence of PDA coating on the release rate, MS (1 mL) and MSCPF (1 mL) were dialyzed against release medium (PBS containing 5% Tween 80, pH = 5.5). At predetermined time points in the above experiments, the amount of released SOR was determined via detecting the changes in UV–vis absorbance at 365 nm.

### Detection of Singlet Oxygen (1O2)

The ^1^O_2_ yield can be measured by using the probe 9,10-anthracenediyl-bis(methylene)dimalonic acid (ABDA) according to a previously reported method. The UV–vis absorption of ABDA at 380 nm gradually decreases with increasing ^1^O_2_ concentration. Specifically, 100 μL of ABDA solution (1.5 mg mL^−1^ in DMSO) was mixed well with different formulations (Ce6 = 3.0 μg mL^−1^) in PBS in the absence or presence of H_2_O_2_ (100 μM). Then, the mixture was irradiated with a 660 nm laser at a power density of 500 mW cm^−2^. The amount of generated ^1^O_2_ was determined by measuring the absorbance change of ABDA at 380 nm at different time points.

### Cellular Uptake

The liver cancer cell line SMMC-7721 was purchased from the Shanghai Institutes for Biological Sciences. The cells were cultured in RPMI-1640 medium supplemented with 10% fetal bovine serum (FBS) and 1% antibiotics (50 unit/mL streptomycin and 50 units/mL penicillin) at 37 ℃ under a humidified atmosphere containing 5% CO_2_.

Flow cytometry analysis was utilized to explore the cellular uptake of the nanoparticles. SMMC-7721 cells were seeded in a 6-well plate (2 × 10^5^ cells/well) and incubated for 24 h. Subsequently, the cells were incubated for 4 h with free Ce6 (8.6 μg mL^−1^), MnO_2_-Ce6@PDA (MCP, containing 8.6 μg mL^−1^ of Ce6), or MnO_2_-Ce6@PDA-PEG-FA (MCPF, containing 8.6 μg mL^−1^ of Ce6). Afterward, the cells were washed with PBS, and digested with trypsin. The cells were collected by centrifugation and then re-suspended in PBS and detected by flow cytometry.

### PDT Efficiency In Vitro

To investigate the biocompatibility of MCPF under dark condition, SMMC-7721 cells were seeded in a 96-well plate (2 × 10^3^ cells/well) and cultured for 24 h. Then, the medium was discarded and replaced with fresh medium containing different concentration of MCPF (0, 2.5, 5, 10, 20, 40, 100, and 200 μg mL^−1^). After 24 h incubation, the cells were washed with PBS and fresh medium containing MTT (0.5 mg mL^−1^) was added. Four hours later, the absorbance was measured at 570 nm using a microplate reader (Thermo Fisher), and the cell viability was analyzed.

For observing the photo-cytotoxicity of MCPF toward cancer cells after laser irradiation, the SMMC-7721 cells were seeded in a 96-well plate and incubated for 24 h. Then, the MCPF (122 μg mL^−1^) or free Ce6 (8.6 μg mL^−1^) were added into the medium with another 6 h incubation. Next, the cells were washed with PBS, and irradiated without or with laser at 808 nm (for PTT, 1.5 W cm^−2^) or 660 nm (for PDT, 500 mW cm^−2^) for 10 min. After 24 h of incubation, the cell viability was measured by using the standard MTT method.

### Measuring ROS Production

For detecting intracellular ROS generation, the cancer cells were incubated with DCFH-DA (10 µM) along with Ce6 (8.6 μg mL^−1^) or MCPF (contained 8.6 μg mL^−1^Ce6) for 2 h, followed by irradiation with a 660 nm laser (500 mW cm^−2^, 10 min). Then, the cells were washed with PBS and nuclei were stained with DAPI. Finally, fluorescence imaging (FL) of DCF cells was analyzed using flow cytometry.

### Apoptosis Analysis

The cell apoptosis was detected using Annexin V-FITC/PI staining method. Briefly, SMMC-7721 cells were seeded in 6-well plate and cultured until a confluent monolayer of cells formed. Then cells were washed and treated with fresh medium containing sorafenib (10 μg mL^−1^), MCPF (122 μg mL^−1^), or MSCPF (159 μg mL^−1^) for 4 h. Afterward, the cells were washed with PBS, irradiated by 660 nm and 808 nm laser for 10 min, and incubated for 24 h. Next, the cells were collected and stained with Annexin V-FITC/PI solution according to the instructions of assay kit (Beyotime, China). Finally, apoptosis-inducing capability was detected by flow cytometry (FCM, BD FACSVerse, USA).

### 5-Ethynyl-2′-Deoxyuridine (EDU) Assay

To assess cell proliferation, SMMC-7721 cells displaying logarithmic growth were seeded into 96-well plates (1 × 10^5^ cells/well) for 24 h. The cells were then treated with fresh culture medium containing SOR (10 μg mL^−1^), MCPF (122 μg mL^−1^), or MSCPF (159 μg mL^−1^) and incubating for 12 h. Subsequently, the cells were exposed to 808 nm and 660 nm laser irradiation (5 min for each). After laser irradiation, the cells were incubated with 50 μM EdU for 2 h, fixed with 4% paraformaldehyde for 30 min, treated with 0.5% Triton X-100, and washed with PBS. Next, 100 μL of 1 × Apollo® reaction cocktail was added to the cells for incubation for 30 min, and subsequently Hoechst 33,342 for cell nuclei staining in the dark. The fluorescence images were captured by a fluorescence microscope (Olympus, Tokyo, Japan), and rate of EdU-positive cell number to the DAPI- positive cell number was quantified using IMAGEJ software.

### Cell Motility

The migration and invasion ability of cells were evaluated using scratch wound-healing and transwell assays. For the scratch wound-healing assay, cells were seeded into a 6-well plate (5 × 10^4^ cells/well) and cultured for 24 h. Then the cells were scraped using a sterile pipette tip and treated with SOR, MCPF, or MSCPF in the appropriate concentration. After 12 h, both MCPF and MSCPF groups were irradiated with a combination of 808 and 660 nm laser for 10 min. Subsequently, cells were washed with PBS, and phase contrast images were taken using NICON Eclipse Ti microscope at 0 and 24 h time points. The distance of cell migration was measured using ImageJ software, and percent of wound healing was calculated.

For the transwell assay, transwell membranes (Corning, NY, USA) coated with Matrigel were applied. Approximately 2 × 10^5^ cells were seeded into the top chamber of a Corning chamber (Corning Pharmingen, San Diego, CA) in serum-free medium containing 0.3% BSA. Complete medium containing 10%FBS was added into the lower chamber. After incubation for 24 h, cells that migrated to or invaded to the underside of the membrane were stained using a 0.1% crystal violet solution (Beyotime). The number of migratory cell was quantified using a light microscope (Nikon Eclipse TE2000-S, Japan). The results were obtained from three independent assays.

### Electron Microscopy Analysis

Twenty-four hours after drug treatment, cells were fixed using 2.5% glutaraldehyde followed by 1% osmium tetroxide. Next, cells were washed with PBS, dehydrated using graded acetone, and embedded in epoxy resin. After slicing by the ultra-microtome, cells were stained using uranyl acetate and lead citrate and observed by transmission electron microscope (S-3400 N, Hitachi, Japan).

### Western Blot

Total proteins were extracted using RIPA lysis buffer (Beyotime Biotechnology, Shanghai, China) and quantified by a BCA protein assay kit. Then equal quantities of proteins were loaded onto a SDS-PAGE gel and transferred onto polyvinylidene difluoride membranes (Millipore, Bedford, Germany) in Tris–glycine buffer. After blocking with 5% fat-free skim milk at room temperature for 1 h, the membranes were incubated with primary antibodies at 4 °C overnight. Subsequently, the membranes were washed and incubated with secondary antibodies at room temperature for 1 h. Finally, the membranes were washed with TBST solution and visualized with an ECL detection system (FDbio, Shenzhen, China). Primary antibodies against Nrf2, GPX4, P-gp were used, with GAPDH as loading controls.

### In Vivo Antitumor Effect of MSCPF NPs

Male BALB/c nude mice (6 weeks old) were purchased from ChangZhou Cavens Laboratory Animal Center (China). Animals received care in accordance with the guidelines for the Care and Use of Laboratory Animals, and the experimental procedures were approved by the Animal Care and Use Committee of Nanjing University of Chinese Medicine.

The tumor-bearing model was developed by subcutaneous injecting SMMC-7721 cells into the armpits of nude mice at a density of 1 × 10^7^ cells/200 µL. When tumor volume reached approximately 100 mm^3^ (set as day 0), the mice were randomly divided into 4 groups (*n* = 6): PBS; SOR (630 μg kg^−1^, i.v); MCPF NPs + 808 nm + 660 nm laser irradiation (dose of MCPF = 7.67 mg kg^−1^, i.v).; MSCPF + 808 nm + 660 nm laser irradiation (dose of SOR = 630 μg kg^−1^; dose of MSCPF = 10 mg kg^−1^, i.v). For the laser treatment groups, laser irradiation was carried out at 24 h post-injection for 10 min. The laser power density was 500 mW cm^−2^ at 660 nm and 1.5 W cm^−2^ at 808 nm. Tumor volumes of the mice were monitored every 2 days. IR images of the mice were recorded with an IR thermal camera. The center of the tumor area was irradiated by an NIR laser (808 nm, 1.5 W cm^−2^) for 10 min, and thermal images were acquired 24 h postinjection with an IR camera. On day 18, the mice were sacrificed and tumor sections were collected.

Tumor tissues were fixed in 4% formalin, paraffin-embedded and sliced into serial Sects. (5 µm thickness). Sections were either analyzed histologically (HE) or stained with antiproliferation antigen antibodies (Ki-67) or a terminal transferase-mediated dUTP nick end-labeling (TUNEL) kit to detect apoptotic cells.

### Intracellular Fe^2+^, LOP, MDA and GSH Measurement

LOP and MDA levels were detected, respectively, using an LOP (BC0025, Solarbio) and MDA detection kit (BC0025, Solarbio) according to the instructions. Glutathione (GSH) content were measured with a micro reduced glutathione determination kit (A006-2–1, Nanjing Jiancheng). Ferro Orange (F-374, Dojindo) was used to detect the intracellular Fe^2+^ level.

### Statistical Analysis

All quantitative results are expressed as the mean ± standard deviation (SD). The statistical analysis was performed by one-way ANOVA using GraphPad Prism (v.8.0) and considered statistically significant when *P* < 0.05.

## Results and Discussion

### Synthesis and Characterization of MnO2-SOR-Ce6@PDA-PEG-FA Nanoparticles

**Fig. 1:**
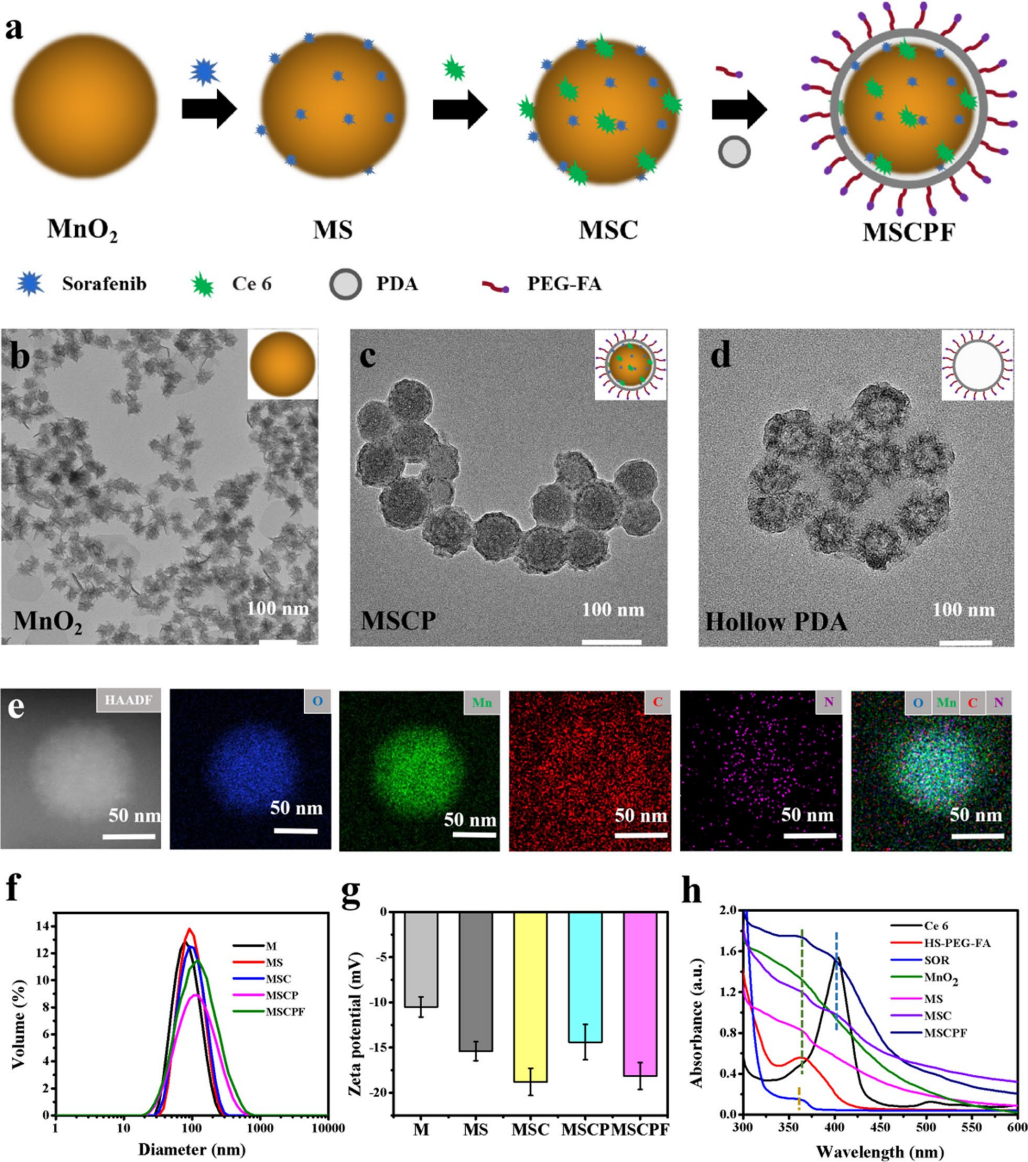
Synthetic procedure and characterization of the nanoparticles. **a** Schematics illustrating the step-by-step synthesis of the MSCPF NPs. **b–d** TEM image of MnO_2_ nanostructure (**b**), the obtained MSCP (**c**) and hollow PDA (**d**). **e** HAADF-STEM image and elemental mapping for MSCP NPs. **f–g** The size distribution and zeta potentials of MnO_2,_ MnO_2_-SOR (MS), MnO_2_-SOR-Ce6 (MSC), MnO_2_-SOR-Ce6@PDA (MSCP) and MnO_2_-SOR-Ce 6@PDA-PEG-FA (MSCPF). **h** The UV–vis absorption of MnO_2_, MS, MSC, MSCP, MSCPF, SOR, Ce6, and HS-PGE-FA

The synthesis schematic diagram of MnO_2_-SOR-Ce_6_@PDA NPs is illustrated in Fig. 1a. First, MnO_2_ NPs were synthesized by a reduction reaction of KMnO_4_ using formamide as reducing agent. Transmission electron microscopy (TEM) (Fig. 1b) and dynamic light scattering (DLS) (Fig. 1f) measurement indicated that MnO_2_ NPs exhibited a three-dimensional hydrangea-like morphology, and uniform size distribution with an average hydrodynamic diameter around 70 nm. The hydrangea-like structure and large specific surface area of MnO_2_ NPs would significantly enhance the loading capacity of drugs and photosensitizers for synergistic cancer therapy. The hydrangea-structured MnO_2_ NPs prepared above were then used as carriers to adsorb anticancer drug sorafenib and the PDT photosensitizer Ce6 to obtain MnO_2_-SOR-Ce6 (MSC) NPs dispersion in aqueous. Since sorafenib and Ce6 are rich in N-containing groups, they can be efficiently adsorbed on the rough MnO_2_ layer through electrostatic interaction and Mn-N coordination. The loading capacity of SOR was calculated through measuring the proportion of residual mass of SOR in the supernatant to the total mass of MS. The loading efficiency and SOR content determined were 74.3 ± 5.6% and 6.3 ± 1.2%, respectively. In addition, the loading efficiency and content of Ce6 were also examined to be 61.3 ± 5.6% and 5.4 ± 0.7%, respectively. These results suggested that sorafenib and Ce6 were both well adsorbed on the pore or surface of MnO_2_ NPs.

To further improve the dispersibility and biocompatibility of MSC NPs, PDA was modified on surface of the nanoclusters through self-polymerization of dopamine in a weak alkaline solution, yielding MnO_2_-SOR-Ce6@PDA (MSCP). Polymerized from dopamine, PDA exhibits excellent adhesive properties and photothermal conversion ability, which provides the adhesiveness to form organic nanoscale thin films with high energy conversion on material surfaces [26]. Additionally, PDA coatings could enhance mucopenetration and cell uptake of nanoparticles and promote hydrophobic drug delivery, making PDA an excellent photothermal agent [27]. Due to its pH-response, MSCP specifically released sorafenib and Ce6 in the tumor microenvironment. After the surface wrapped with PDA, TEM images indicated that a uniform thin shell was formed on the surface of the MSCP NPs, and the contrast of the sample was significantly deepened (Fig. 1c). The remaining hollow PDA shell was observed when the MSC core was sequentially removed in H_2_O_2_ solution and Na_2_CO_3_ aqueous solution (Fig. 1d). Moreover, the hydrodynamic diameter of MSCP NPs increased to 117.09 ± 5.38 nm, and the Zeta potential changed to − 14.16 ± 2.47 mV, thus confirming the successful modification of PDA (Fig. 1d and e). In addition, element mapping was performed to confirm the formation of MSCP (Fig. 1g). In order to prolong the circulation lifetime of nanoparticles in vivo, HS-PEG-FA with targeting effect was functionalized on the surface of MSCP NPs, obtaining MnO_2_-SOR-Ce6@PDA-PEG-FA (denoted as MSCPF). PEG-FA can effectively prolong the circulation retention and enhance the effective aggregation of MSCPF NPs in the liver, realizing a synergic tumor-targeted chemo-photothermal therapy. During the synthesis process, the gradual change in the size, surface potential, and UV–vis absorption of nanoparticles indicated the successful preparation of MS, MSC, MSCP, and MSCPF (Fig. 1f–h).

### Photothermal Effect of MnO2-SOR-Ce6@PDA-PEG-FA (MSCPF)

**Fig. 2:**
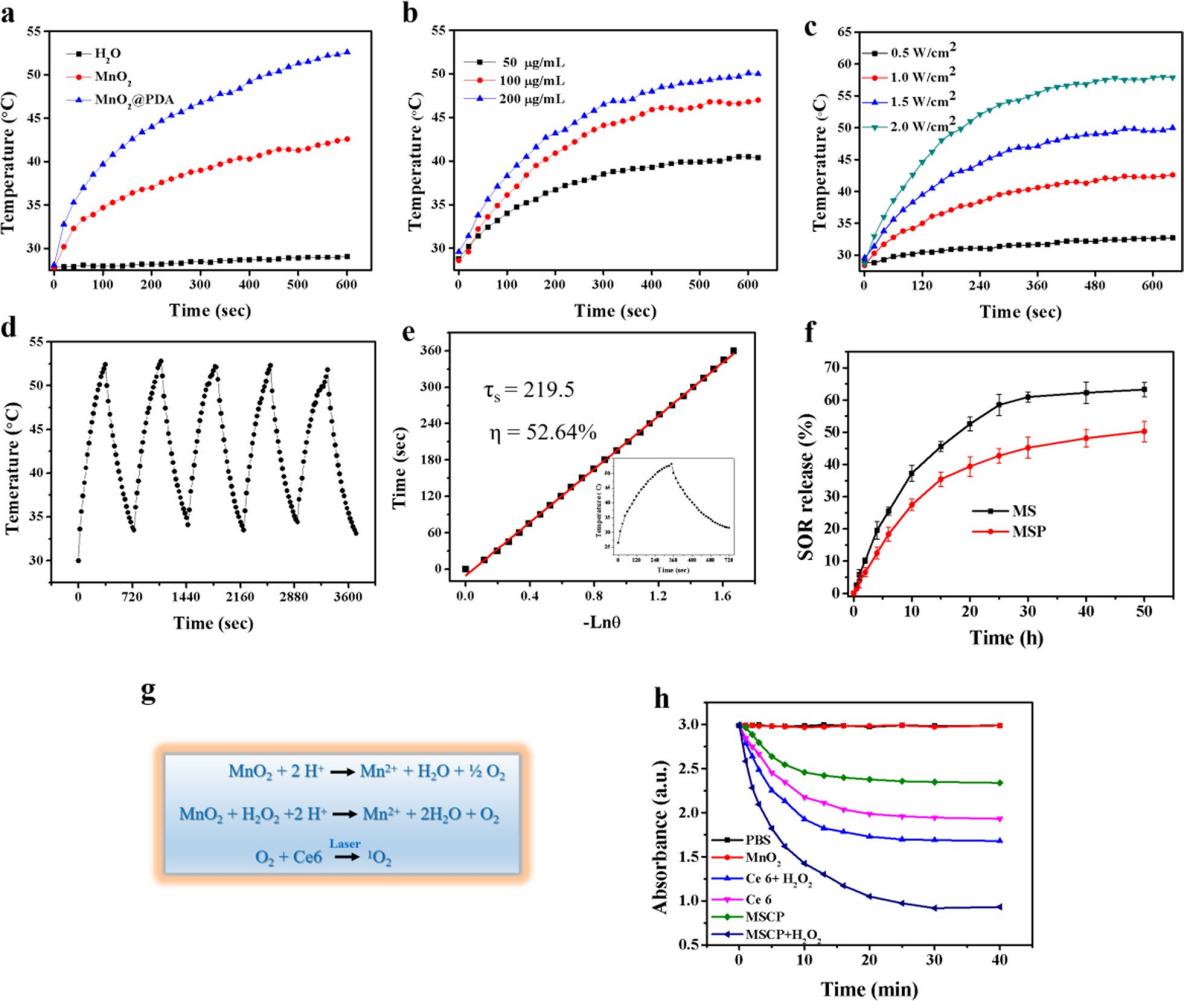
**a** Temperature increase versus irradiation duration curves of H_2_O, MnO_2_ (100 μg mL^−1^), and MnO_2_@PDA (100 μg mL^−1^) at irradiation with an 808 nm laser (2 W cm^−2^). **b** Temperature increase versus irradiation duration curves of MSCPF at different doses (50, 100, 200 μg mL^−1^) at power density of 1.5 W cm^−2^. **c** Temperature increase versus irradiation duration curves of MSCPF (200 μg/mL) at different power density (0.5, 1.0, 1.5, 2.0 W cm^−2^). **d** Heating/cooling profiles of MSCPF after five on–off laser irradiation cycles (808 nm, 1.5 W/cm^2^). **e** Linear data of cooling time versus driving force temperature for MSCPF, with calculated PCEs of 52.64%. **f** Triggered release profiles of SOR from MnO_2_-SOR, or MnO_2_-SOR @PDA in Tris–HCl solution containing 5% Tween 80 and 100 μM H_2_O_2_ (pH 5.5). **g** Schematic diagram of singlet oxygen produced by MnO_2_. **(h)** ROS production profiles of PBS, MnO_2_, Ce6, MSCP, Ce6 + H_2_O_2_, MSCP + H_2_O_2_ under laser irradiation (660 nm, 500mW cm^−2^) measured by UV–vis with 9,10-anthracenediyl-bis(methylene) dimalonic acid (ABDA) as a ROS probe

To test the photothermal properties of MnO_2_ and PDA, the photothermal conversion effects of H_2_O, MnO_2_, and MnO_2_@PDA were evaluated by irradiation with an 808 nm laser (2 W cm^−2^) for 10 min (Fig. 2a). In comparison with H_2_O, MnO_2_ showed a significant temperature rise from 28.9 to 42.7 °C, while MnO_2_@PDA showed higher photothermal conversion efficiency, with temperature rising from 28.9 to 53.1 °C in 10 min. Due to the high absorption in the NIR-II window, PDA coating could enhance the photothermal efficiency of MnO_2_. Next, the photothermal conversion capability of MSCPF were determined. A serious of MSCPF solution (50, 100, 200 μg mL^−1^) or different power density (0.5–2.0 W cm^−2^) were applied for test, and the temperature rise was recorded by NIR thermal camera. As shown in Fig. 2b and c, the photothermal effect of MSCPF was dependent on both concentration and laser-power, indicating the heat generation could be neatly tuned. Meanwhile, MSCPF showed no significant temperature decay within five cycles of laser irradiation, demonstrating excellent yet stable light-to-heat conversion property (Fig. 2d). The photothermal conversion efficiency (PCE) of MSCPF at 808 nm and power density of 1.5 W cm^−2^ was calculated to be 52.64% according to the published method (Fig. 2e) [25]. Taken together, the excellent photothermal capabilities of MSCPF make it potentially useful for photothermal therapy.

### Drug Release and Singlet Oxygen Generation of MSCP NPs

Loading of sorafenib on MnO_2_ NPs facilitates the sustained drug release. The release process of sorafenib from MnO_2_/SOR (MS) and MnO_2_-SOR@PDA (MSP) was monitored by UV–vis absorption spectroscopy in the presence of H_2_O_2_. Due to the degradation of MnO_2_ in the acidic solution containing H_2_O_2_, sorafenib could be sustained released from MS NPs, and the coated PDA can effectively suppress burst drug release (Fig. 2f). It is well recognized that MnO_2_ could catalyze H_2_O_2_ to produce O_2_ and reverse the tumor hypoxia (Fig. 2g) [28]. Moreover, photosensitizers Ce6 could generate cytotoxic singlet oxygen for killing tumor cells upon irradiation [29]. With the ability to generate singlet oxygen for MSCP NPs in the presence of H_2_O_2_, the amount of ^1^O_2_ was investigated using a ^1^O_2_ probe, 9,10-anthracenediyl-bis(methylene) dimalonic acid (ABDA), whose absorbance at 380 nm could be reduced by^1^O_2_. Obviously, the absorbance of Ce6 solution decreased from 3.02 to 1.97 over 25 min without H_2_O_2_, and further to 1.71 after H_2_O_2_ addition (100 μM) (Fig. 2h). For MSCP, the absorbance decreased rapidly from 3.02 to 0.92 within 25 min under H_2_O_2_ condition, which is due to the large amount of ^1^O_2_ production. By contrast, owning to the quenching effect of MnO_2_ on Ce6, ^1^O_2_ yield of MSCP was lower than that of free Ce6 without H_2_O_2_. These results indicated that. MSCP had a great potential in alleviating tumor hypoxia through O_2_ generation, thereby enhancing the anticancer effect of PDT.

### Cellular Uptake and Phototherapeutic Efficiency of MCPF NPs

**Fig. 3:**
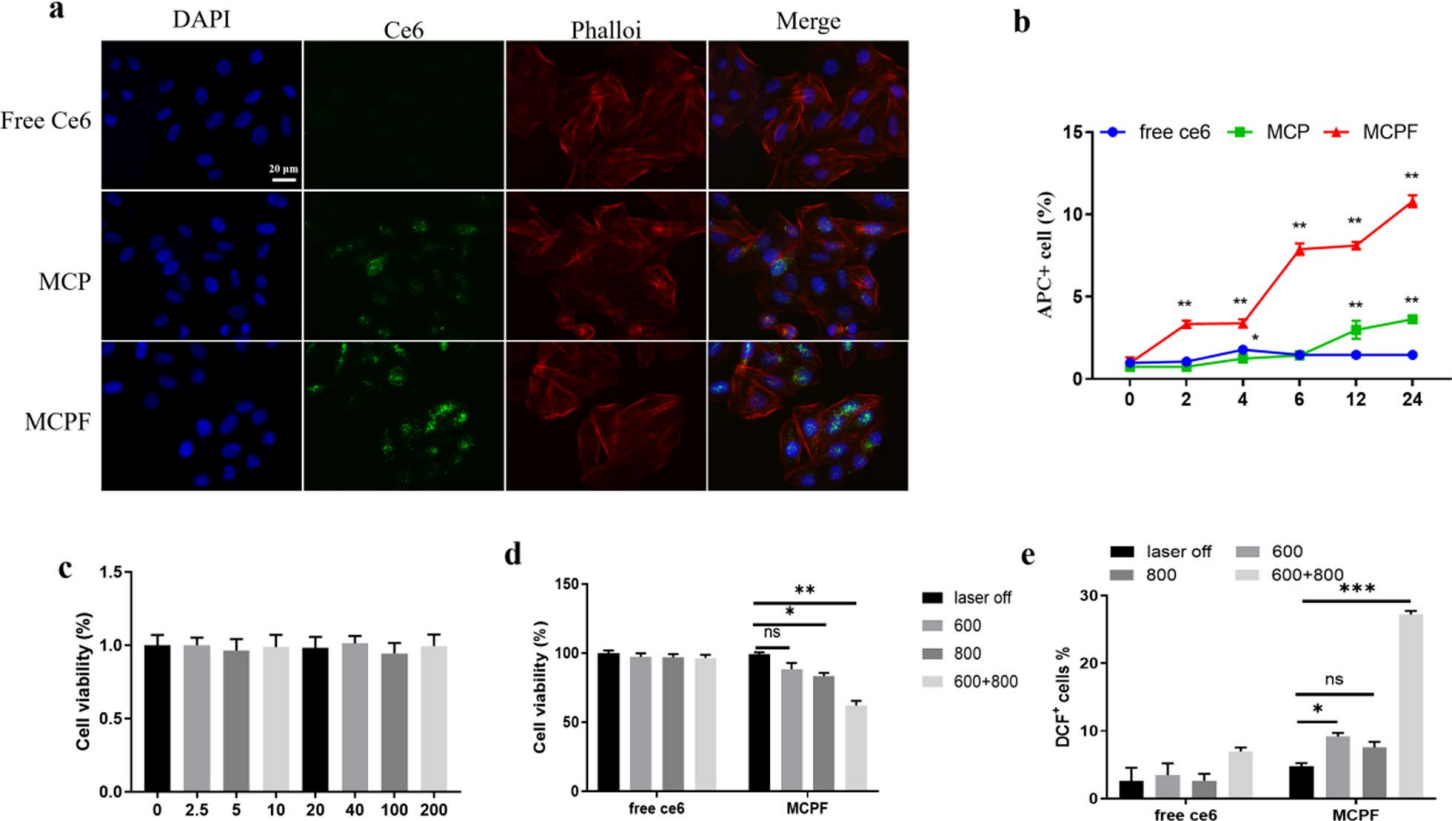
**a–b** Confocal fluorescence images (**a**) and flow cytometry data (**b**) of SMMC-7721 cells after incubation with free Ce6, MCP, and MCPF for 4 h. **c** The cytotoxicity of MCPF on SMMC-7721 cells without laser irradiation. **d** Cell viability of SMMC-7721 cells treated with free Ce6 or MCPF under single or combined laser irradiation (660 nm at 500 mW cm^−2^ and 808 nm at 1.5 W cm^−2^). **e** The ROS-producing capability of MCPF NPs detected by flow cytometry using DCFH-DA as an indicator

To assess the cellular uptake behaviors, SMMC-7721 cells were incubated with free Ce6, MCP, and MCPF, followed by flow cytometry analysis. As shown in Fig. 3a and b, cells receiving MCP showed greater Ce6 fluorescence than those treated with free Ce6, reflecting the enhancement of cellular uptake by nanodelivery. In particular, the highest Ce6 signal was observed in cells incubated with MCPF, which was due to the enhanced delivery efficiency by FA through the active targeting effect.

Good bio-safety is an essential prerequisite for nanocarriers [30]. Inspired by the excellent uptake of MCPF by cells, we further examined the cytotoxicity of MCPF in the dark in vitro. MTT assay indicated that MCPF exhibited no obvious toxicity to SMMC-7721 cells even at high dosage (200 μg mL^−1^), suggesting the good biocompatibility of MCPF NPs (Fig. 3c). To further evaluate the enhanced phototherapeutic efficiency of MCPF, SMMC-7721 cells were incubated with Ce6 or MCPF and exposed to 660 nm (500 mW cm^−2^) light for PDT and/or 808 nm (1.5 W cm^−2^) light for PTT. Twenty-four hours later, cell viability was assessed. As shown in Fig. 3d, MCPF showed high phototoxicity on cell viability and exhibited a synergistic effect at simultaneous laser irradiation (660 nm and 808 nm), suggesting better antitumor effect of PTT combined PDT. In case of free Ce6, the degree of phototoxicity was negligible on cancer cells under dark condition or irradiation. These results indicated that the phototherapy effect of MCPF could be controlled by light excitation, which offered the possibility for selective treatment of cancers with minimal side effects.

Effective light-generated ROS was cytotoxic in tumor cells, which could induce cell apoptosis and death [31]. To evaluate the ROS-producing capability of MCPF NPs, intracellular ROS was detected by flow cytometry using DCFH-DA as an indicator. As observed in Fig. 3e, both free Ce6 and MCPF groups showed weaker fluorescence intensity without laser treatment. When exposed to laser irradiation, SMMC-7721 cells treated with MCPF showed a considerable increase in the fluorescence, and the strongest intense fluorescence was observed under simultaneous laser irradiation (660 nm and 808 nm). Quantitative analyses revealed a 3.90-fold higher ROS production in the MCPF -treated cells than free Ce6-treated ones (Fig. 3d), demonstrating that MCPF could effectively produce large amounts of ROS under light irradiation. This data is well consistent with the above-mentioned ^1^O_2_ production and the PDT effect by MCPF, further confirming that the nanoplatform is capable of executing its PDT function within the cells.

### In Vitro Synergistic Chemo/PTT/PDT Effects of MSCPF NPs

**Fig. 4:**
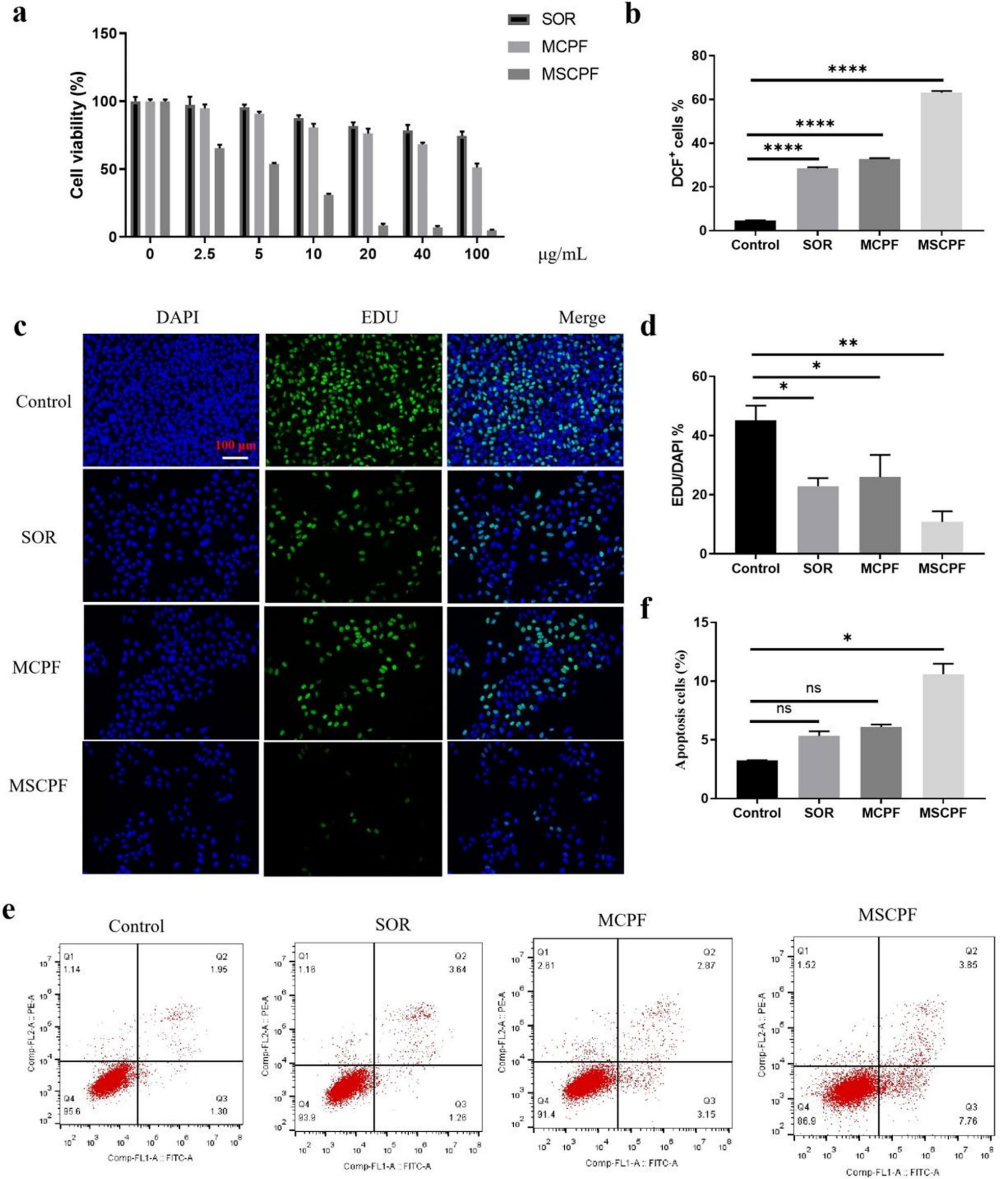
**a** In vitro cytotoxicity of different concentrations of MSCPF NPs on SMMC-7721 cells. **b** ROS production of MSCPF nanoplatform within the SMMC-7721 cells upon irradiation (660 nm at 500 mW cm^−2^ and 808 nm at 1.5 W cm^−2^). **c** Representative images of cell proliferation by 5-ethynyl-2′-deoxyuridine (EDU) assay in vitro. **d** The percentage of EdU-positive cells were quantified. **e** Apoptosis of cancer cells were analyzed by Annexin V-FITC/PI staining by SOR, MCPF, and MSCPF. **f** The apoptosis rates of SMMC-7721 cells were represented

As it has been reported, combined PTT/PDT could increase the sensitivity of cancer cells to chemotherapy, thereby promoting the ability to overcome drug resistance [32]. Next, we detected the cytotoxicity of MSCPF against SMMC-7721 cells in vitro. Sorafenib against SMMC-7721 cells was first tested, and no apparent cytotoxicity was found with an increasing concentration ranging from 2.5 to 100 μg/mL (Fig. 4a). This observation is reasonable due to the low intracellular accumulation of free sorafenib. Owing to the synergistic combined PTT/PDT effects MCPF exhibited a significantly improved inhibition efficacy on SMMC-7721 cells in comparison with sorafenib. Notably, MSCPF exhibited a remarkable enhanced cell-growth inhibition compared to free sorafenib or MCPF, demonstrating the synergistic anticancer effect of combination therapy (Fig. 4a). Subsequently, the capacity of MSCPF to produce ROS upon irradiation (660 nm and 808 nm) was measured. As expected, MSCPF showed stronger fluorescent intensity than sorafenib or MCPF, implying that MSCPF could produce high level of cellular ROS (Fig. 4b). The EdU assay shown in Fig. 4c and d further verified that MSCPF induced lower EdU-positive cell rate than sorafenib or MCPF, as was attributed to the combination of PDT/PTT and chemotherapy. Additionally, due to ROS generation and sorafenib release, MSCPF treatment induced higher apoptosis rate (10.6%) than the single chemotherapy (5.34%) or single photo-therapeutic group (6.11%) (Fig. 4e and f).

**Fig. 5:**
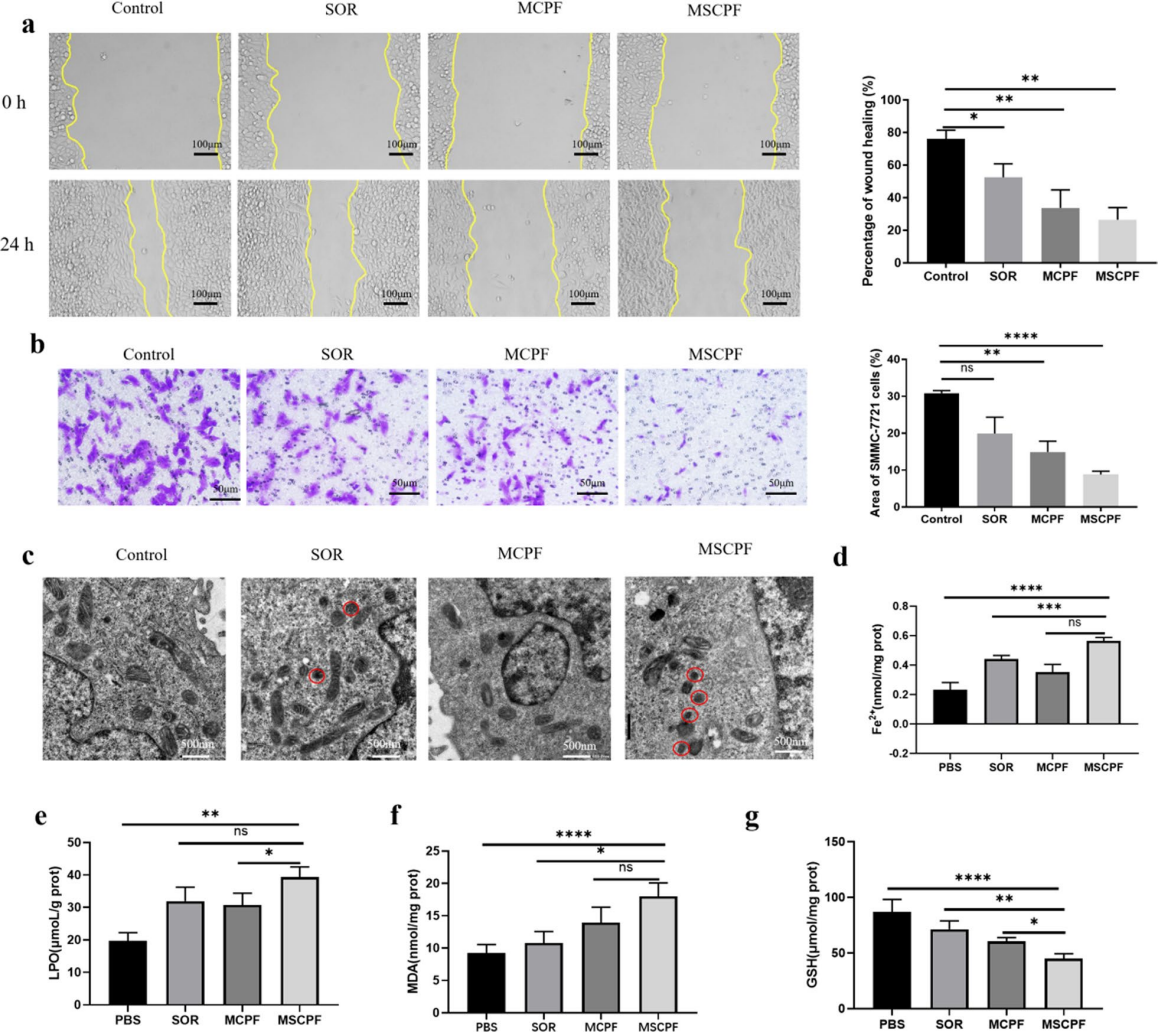
**a** Cell migration detected by wound healing assay. The statistical graph (right) shows the corresponding cell migration rate. **b** Images of cell invasion by transwell assay. **(c)** TEM observations of the mitochondria morphology of SOR, MCPF, and MSCPF corresponding to the in vitro studies. **d–g** Intracellular content of free Fe^2+^, lipid peroxidation (LPO), malondialdehyde (MDA), and glutathione (GSH) in SMMC-7721 cells treated by SOR, MCPF, and MSCPF (660 nm irradiation at 500 mW cm^−2^ and 808 nm irradiation at 1.5 W cm^−2^)

It is reported that resistant cells display enhanced migration and invasion ability. Hence, we investigated whether MSCPF NPs could further enhance the inhibition of cell migration using wound-healing and transwell assays. Regarding the wound healing assay, cells in the untreated and sorafenib-treated groups filled the wound largely after culture for 24 h, while MCPF and MSCPF groups exhibited reduced migration into the wound (Fig. 5a). The wound healing rate of MSCPF-treated group significantly decreased compared with those in control or sorafenib-treated groups. Meanwhile, transwell assays showed that invasion of SMMC-7721 cells after sorafenib treatment was lower than that in control (*P* > 0.05). The invasion of cells in MSCPF decreased to 8.83% compared with those (19.9%) in sorafenib alone (Fig. 5b). In brief, MSCPF could induce a significant combination of chemo/PTT/PDT effects, namely not only decreasing cell motility but also abolishing the invasion of cancer cells.

## MSCPF NPs Enhanced Antitumor Efficacy by Inducing Ferroptosis and Inhibiting P-gp Expression

**Fig. 6:**
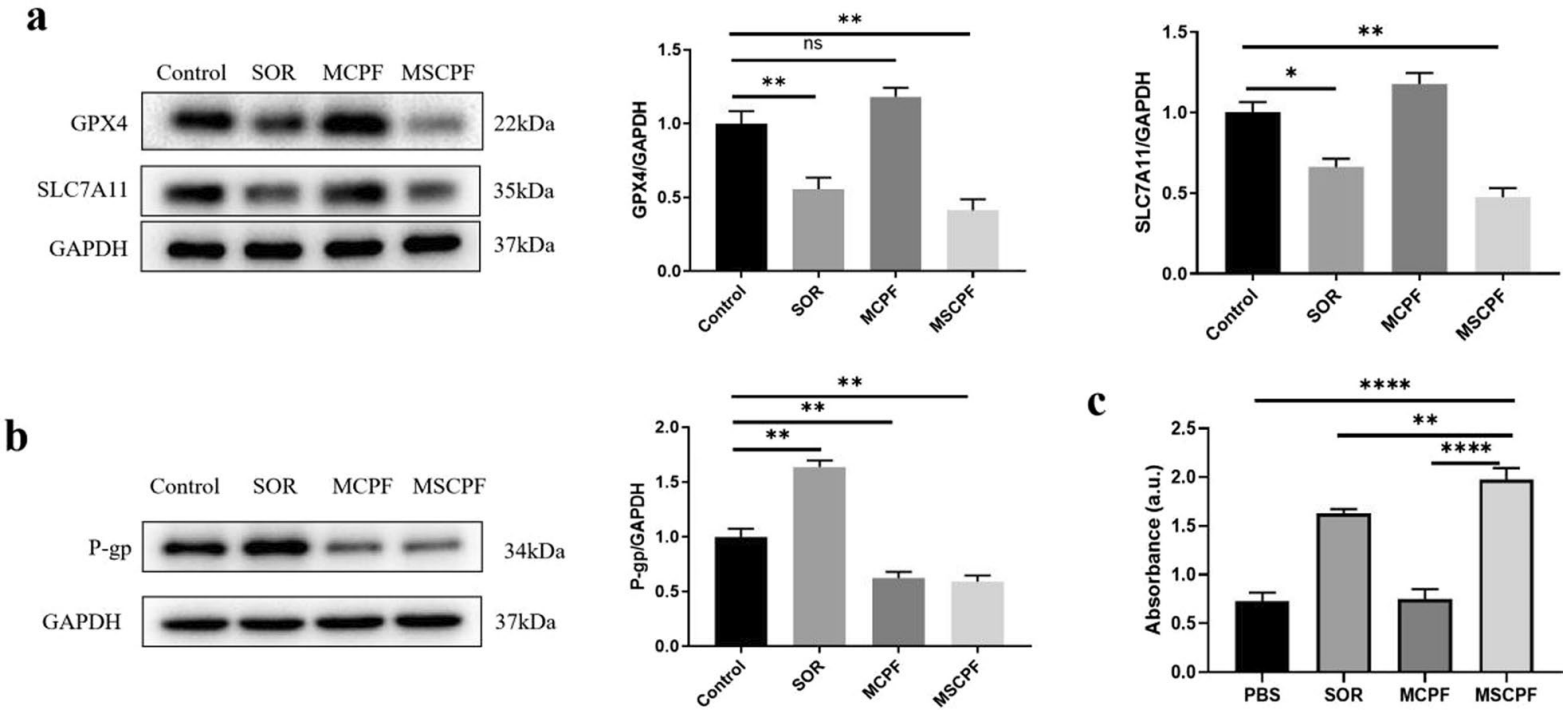
**a** WB assay and quantitative analysis of the expression of GPX4 and SLC7A11 in vitro studies. **b** WB assay and quantitative analysis of the expression of P-gp in SMMC-7721 cells treated by PBS, SOR, MCPF, or MSCPF (660 nm irradiation at 500 mW cm^−2^ and 808 nm irradiation at 1.5 W cm^−2^). **c** Concentration assay of sorafenib in cells after SOR, MCPF and MSCPF treatment using Uv-vis spectrophotometry

To explore the underlying mechanism of enhanced combination therapy by MSCPF, the microstructures of tumor cells were examined by TEM. As displayed in Fig. 5c, in comparison with the control group, MSCPF-treated cells showed smaller mitochondria, loss of mitochondrial ridges and increased membrane density, all of which are typical morphological changes of ferroptosis [33]. Ferroptosis could be triggered by iron-dependent accumulation of lipid peroxides [34]. To further validate ferroptosis, the levels of free Fe^2+^, lipid peroxidation (LPO), and malondialdehyde (MDA, the end product of LPO) were examined with the corresponding assay kits. The measurement results suggested that, compared with the control and sorafenib groups, the levels of free Fe^2+^, LOP, and MDA were significantly higher in the MSCPF-treated group (Fig. 5d–f). Meanwhile, several evidence has revealed that glutathione peroxidase 4 (GPX4) utilizes glutathione (GSH) to detoxify lipid peroxidation and acts a central repressor of ferroptosis. The activity of GPX4 depends on SLC7A11, which is a crucial protein regulating the synthesis of GSH that functions to inhibit LPO production. As shown in Figs. 5g and Fig. 6a, MSCPF NPs treatment significantly down-regulated GPX4, SLC7A11, and GSH expression. Together, these findings indicate that MSCPF could induce ferroptosis to enhanced antitumor efficacy, which is associated with inhibition of SLC7A11/GPX4 axis and altered GSH homeostasis.

P-gp overexpression in cancer cells has been regarded as a major obstacle for chemotherapy. As an ATP-dependent drug efflux pump, P-gp actively expels chemodrugs, such as sorafenib, out of cells, leading to reduced anticancer activity. It has been reported that oxygen production by PDT could efficiently decrease the P-gp expression and then enhanced chemotherapy efficiency [35]. To further understand the potential mechanism by which MSCPF enhances sensitivity of SMMC-7721 cells to sorafenib, P-gp level was measured via western blotting. As predicted in Fig. 6b, in the case of sorafenib group, the expression of P-gp showed significant increase compared to the control group. In sharp contrast, both MCPF and MSCPF significantly inhibited the P-gp expression. Furthermore, the concentration of sorafenib was found to be significantly up-regulated after MSCPF treatment, in line with the western blotting results (Fig. 6c). Taken together, we deduced that MSCPF nanosystem plays a major role in inhibiting P-gp expression, thus leading to increased intracellular accumulation of sorafenib and enhanced antitumor efficiency.

### In Vivo Anti-Tumor Effect of MSCPF NPs

**Fig. 7:**
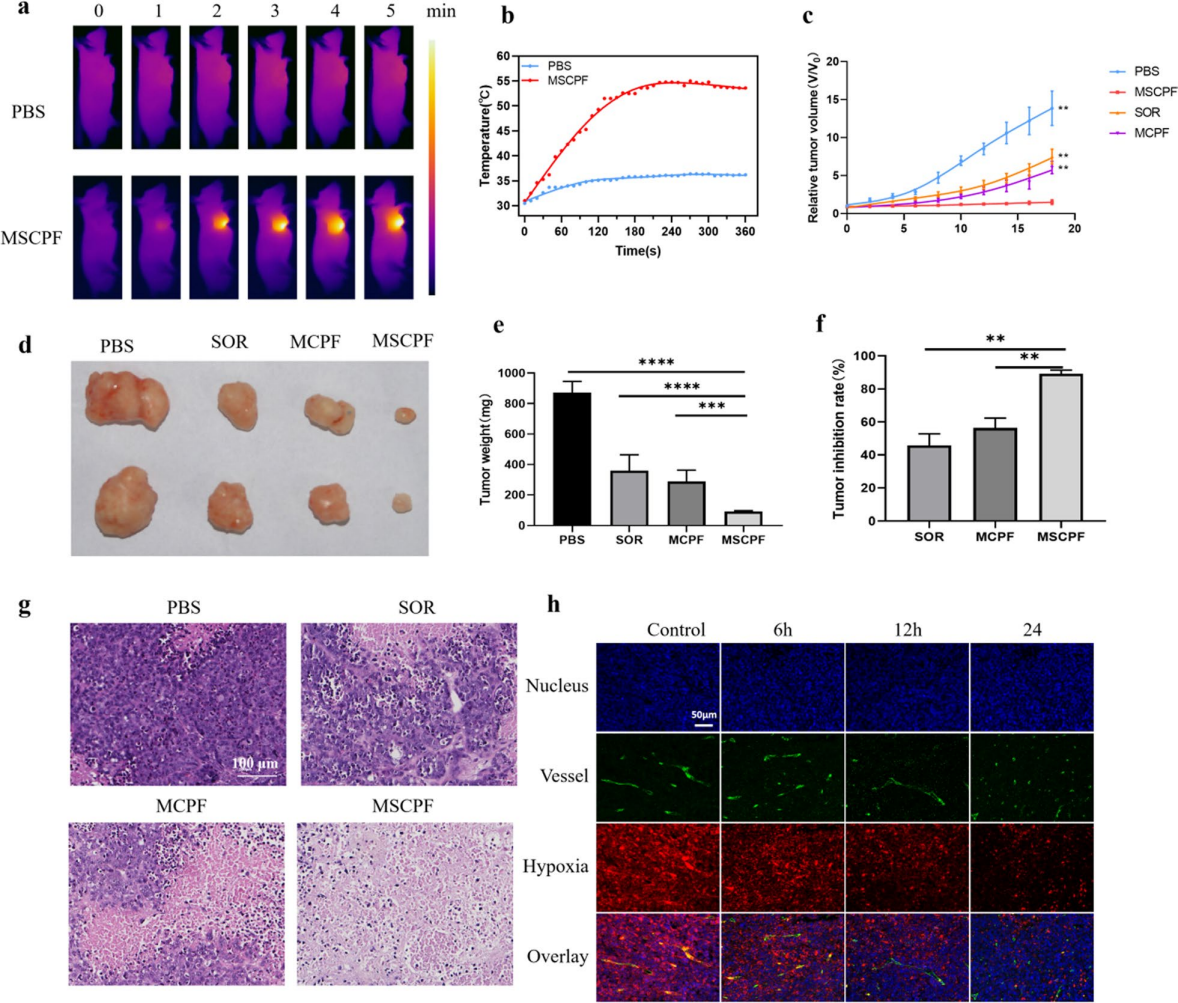
In vivo anti-tumor effect of MSCPF NPs. **a** IR thermal imaging by exposing tumors of mice to irradiation of 808 nm at 1.5 W cm^−2^ for 10 min after injection of PBS or MSCPF. **b** The temperature increase curve of tumors in the PBS and MSCPF groups after exposure to 808 nm irradiation. **c** Tumor growth curves of the mice during the experiment. **d** Photographs of the tumors harvested from mice at the end of treatment. **e** The weight of the tumors was measured. **f** The tumor inhibition rate of PBS, SOR, MCPF, or MSCPF on mice bearing SMMC-7721 tumors. **g** Representative HE staining histological sections of mouse tumors. **h** Immunofluorescence images of tumor slices of mice in control and the MSCPF-treated group after 6, 12, and 24 h injection. The nucleus, vessels, and hypoxic areas were respectively stained with 4′,6-diamidino-2-phenylindole (DAPI) (blue), anti-CD31 antibody (green), and anti-pimonidazole antibody (red)

Encouraged by the superior tumor accumulation of sorafenib, the therapeutic potential of MSCPF was evaluated in vivo. Mice bearing SMMC-7721 tumors were divided into four groups: (i) PBS (control); (ii) sorafenib (SOR); (iii) MCPF + irradiation at 660 and 808 nm, and (iv) MSCPF + irradiation at 660 and 808 nm (concentration of sorafenib = 630 μg/kg). The tumors in the PBS and MSCPF groups were exposed to 808 nm irradiation, and the temperature was recorded by infrared thermography (Fig. 7a). As presented in Fig. 7b, the temperature in tumor tissues of the MSCPF group increased from 31.0 to 54.8 °C within 4 min after tumor-specific light irradiation, which could allow sufficient photothermal effect for tumor ablation. In contrast, there was only a slight temperature increase to 36.0 °C in the PBS group after irradiation. The tumor size of mice was recorded every two days (Fig. 7c). On day 18, the mice were sacrificed, and the tumors were excised, photographed, and weighed (Fig. 7d and e). It was found that the tumor volume increased gradually in the control group, both sorafenib and MCPF with laser irradiation displayed moderate inhibition on tumor growth. For MSCPF-treated group, the tumor growth was significantly suppressed. Compared to the saline-treated control group, sorafenib, MCPF and MSCPF administration caused a 45.80% (*P* < 0.01), 56.30%, and 89.15% inhibition of the tumor volume, respectively (Fig. 7f). Furthermore, the histological tissue images of the tumors also demonstrated the high therapeutic efficacy of MSCPF (Fig. 7g). A large amount of cell death or necrosis was observed in mice treated with MSCPF. However, in mice treated with sorafenib or MCPF, cell death was relatively rare. In addition, tumor sections from mice treated with MSCPF showed a marked decrease in green fluorescence over time, indicating that the MnO2 was effective in reducing tumor hypoxia (Fig. 7h).

**Fig. 8:**
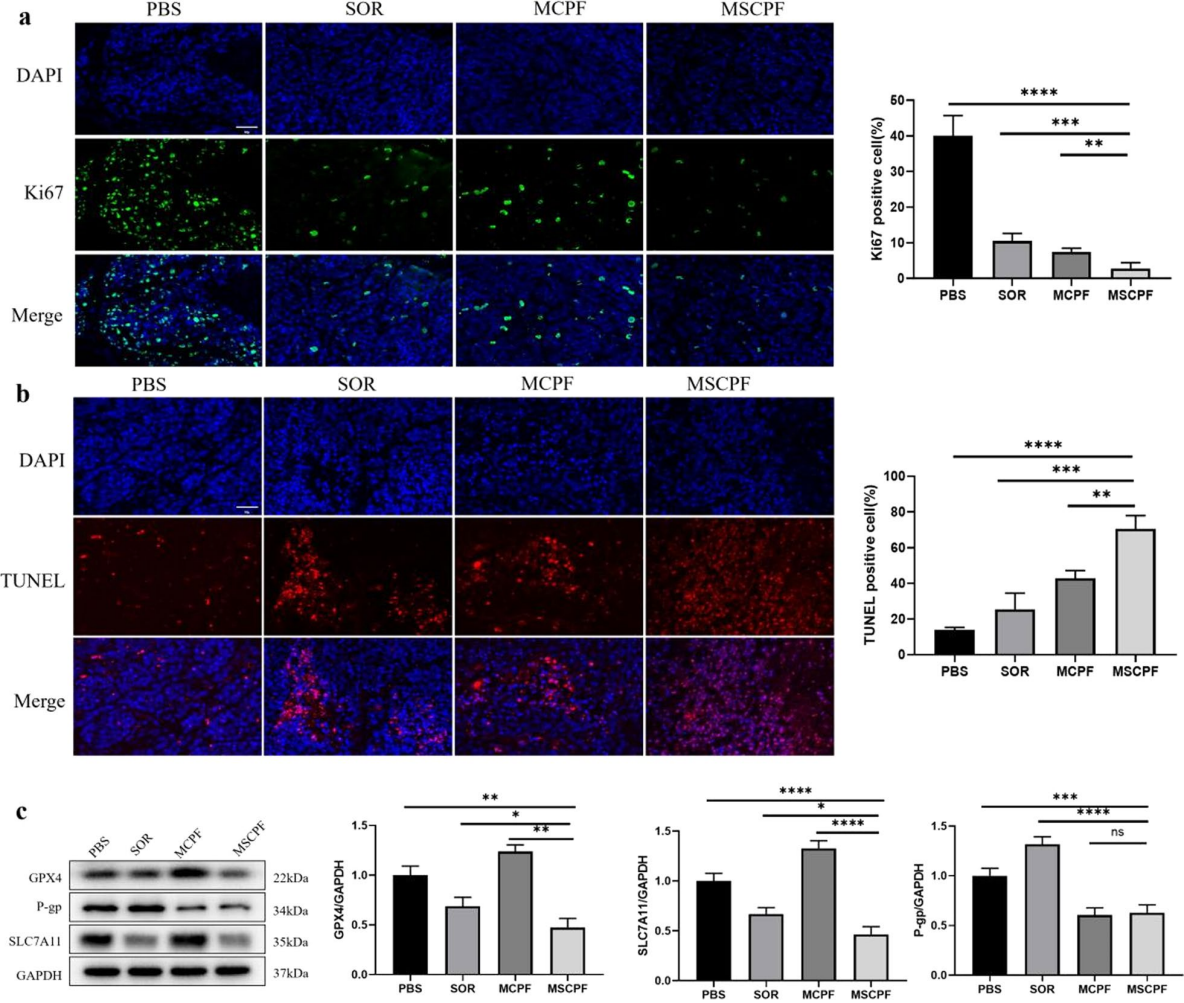
**a** Ki67 staining of the tumor tissue of mice treated with PBS, SOR, MCPF, or MSCPF upon irradiation. **b** TUNEL staining of the tumor tissue of mice treated with PBS, SOR, MCPF, or MSCPF upon irradiation. **c** GPX4, SLC7A11 and P-gp expressions in SMMC-7721 xenograft models treated by PBS, SOR, MCPF, or MSCPF upon irradiation

MSCPF treatment also exhibited reduced expression of Ki-67 proliferation marker (Fig. 8a) and increased terminal deoxynucleotidyl transferase dUTP nick end labeling (TUNEL) staining (Fig. 8b), implying reduced cancer cell proliferation and activation of apoptosis. Further study on the possible mechanism underlying action of MSCPF on tumor growth was performed by detecting key ferroptosis repressors as well as P-gp expression. In line with the in vitro assays, MSCPF significantly reduced the protein levels of GPX4, SLC7A11 and P-gp in SMMC-7721 xenograft models (Fig. 8c). Overall, these in vivo results demonstrated that MSCPF has superior combined treatment effect on tumor growth. The enhanced therapeutic efficacy may not only originate from the high accumulation and singlet oxygen production of MSCPF in tumor tissue, but also from ferroptosis induction and P-gp inhibition.

## Conclusions

In conclusion, we reported a synergistic tumor-targeted and hypoxia-alleviated nanoplatform (MSCPF) that co-deliver sorafenib, Ce6, and MnO_2_ for combined chemo/PDT/PTT therapy to achieve enhanced antitumor effect in HCC. MSCPF NPs can effectively catalyze the decomposition of H_2_O_2_ inside tumors into O_2_, exhibiting good potential in alleviating tumor hypoxia to enhance PDT. Furthermore, strong heat generation and high ROS production mediated by MSCPF were detected upon dual-wavelength laser irradiation. Importantly, MSCPF could facilitate tumor accumulation and distribution of sorafenib by substained drug controlled release and reducing drug efflux via inhibiting P-gp expression. The deactivation of GPX4 and SLC7A11 caused by MSCPF also induces ferroptosis, thus greatly strengthens the antitumor effect. Both cell and animal experiments demonstrated that the cancer cells and tumor growth were notably inhibited by MSCPF. Collectively, MSCPF constructed in this work shows great potential as a versatile nanoplatform capable of improving cancer therapy efficiency and reducing drug resistance.

## Data Availability

The datasets supporting the conclusions of this article are included within the article.

## Abbreviations

HCC: Hepatocellular carcinoma
Ce6: Chlorine6
MSCPF: MnO2-SOR-Ce6@PDA-PEG-FA
MDR: Multidrug resistance
ABC: ATP-binding cassette
PDT: Photodynamic therapy
PTT: Photothermal therapy
MnO_2_: Manganese dioxide
PDA: Polydopamine
H_2_O_2_: Hydrogen peroxide
EDU: 5-Ethynyl-2′-deoxyuridine
SOR: Sorafenib
GSH: Glutathione
SD: Standard deviation
TEM: Transmission electron microscopy
DLS: Dynamic light scattering
MS: MnO2-SOR
MSC: MnO2-SOR-Ce6
MSCP: MnO2-SOR-Ce6@PDA
